# Replicating and extending research on sanctification: A cognitive appraisal with implications for behaviors, attitudes, and self-image

**DOI:** 10.1016/j.ijchp.2025.100566

**Published:** 2025-05-03

**Authors:** Elizabeth J. Krumrei-Mancuso, Janet P. Trammell, Jennifer A. Harriger, Joshua A. Evans

**Affiliations:** Pepperdine University, United States

**Keywords:** Sanctification, Spirituality, Religion, Physical activity, Exercise, Nature, Access to nature, Body image

## Abstract

Basic competency in religious and spiritual issues among mental health professionals includes knowledge about the role of religion/spirituality in people's lives, particularly as it relates to mental health. This research focuses on cognitive appraisals of sanctification by which individuals interpret stimuli to be sacred. We examined the extent to which adults in the U.S. (*N* = 342) perceived the body, physical activity, and nature as sacred, and how these views related to indicators of mental and physical health. The results indicated that sanctification of the body was associated with greater body appreciation and self-esteem. Sanctification of physical activity was associated with more engagement in physical activity and receiving more enjoyment from physical activity. Finally, sanctification of nature was associated with experiencing greater connectedness to nature and pursuing more nature exposure. We observed a number of moderators among these links. We discuss implications for mental health professionals.

## Introduction

The great lesson from the true mystics, from the Zen monks, and now also from the-Humanistic and Transpersonal psychologists-that the sacred is in the ordinary, that it is to be found in one’s daily life, in one’s neighbors, friends, and family, in one’s back yard…

-Abraham H. Maslow (1964/2021, p. viii)

Most professional boards and associations governing clinical psychologists promote the ethical requirement of basic competency in religious and spiritual issues in mental health. Spiritual competency involves knowledge, skills, and attitudes necessary for effective mental health care (Pargament, 2007). This is important, given recent meta-analytic evidence suggesting that clients who identify as religious or spiritual benefit more from psychotherapy that incorporates a focus on religious/spiritual issues than from psychotherapy that neglects this domain of life (Bouwhuis-Van Keulen et al., 2024). Although it is not relevant to the field of psychology to address the ontological reality of people's religious/spiritual beliefs, the field is concerned with people's cognitions about religion/spirituality and how these cognitions relate to mental and physical health (Pargament & Mahoney, 2005).

For better (Abdel-Khalek et al., 2019; Garssen et al., 2021; Shattuck & Muehlenbein, 2020) or worse (Bockrath et al., 2022), religiousness/spirituality can impact mental and physical health. One way in which religiousness/spirituality play out in people's lives is through their perceptions of sacredness. The subjective interpretations individuals form about the stimuli they encounter have powerful implications for mental health (Ellis, 1957) and physical health (Denson et al., 2009). Such interpretations can be referred to as cognitive appraisals, or simply, appraisals. A particular constellation of appraisals of interest in the psychology of religion and spirituality is referred to as sanctification. Sanctification is the cognitive interpretation that an aspect of life is sacred or holy (Mahoney et al., 1999).

A recent meta-analysis of 63 studies indicated that greater sanctification was associated with better psychological adjustment and less negative functioning with small-to-medium effects (Mahoney et al., 2022). Further, sanctification can be an organizing and guiding force in people's lives, given that individuals invest more time, resources, energy, emotion, and perseverance into aspects of life they sanctify (Pargament et al., 2017).

### Sanctification of the body

One of the commonly studied targets of sanctification is the body. Research has examined link between sanctifying one's body and health habits such as exercise (Homan & Boyatzis, 2010; Mahoney et al., 2005), sleep (Kopp et al., 2017), eating (Goulet et al., 2017; Mahoney et al., 2005), use of alcohol and substances (Mahoney et al., 2005), and stress management (Homan & Boyatzis, 2010). The majority of links have consisted of small, positive relationship between sanctifying one's body and beneficial health outcomes. However, there are exceptions. For example, although some studies have linked body sanctification to better scores on global measures of health-protective behaviors (Mahoney et al., 2005), other studies have observed no links (Benjamins et al., 2011) and even negative links (Ellison et al., 2008) between sanctification of the body and preventative health care activities, such as having an annual check-up.

Of particular interest to the current study, research has examined links between sanctifying one's body and attitudes and beliefs surrounding one's appearance, such as body connectedness, body satisfaction, body appreciation, and body esteem (Goulet et al., 2017; Harriger et al., 2024; Homan & Boyatzis, 2009; Jacobson et al., 2013, 2016a, 2016b; Kusina & Exline, 2021; Mahoney et al., 2005), investment in external appearance (Goulet et al., 2017), body shame and body shape preoccupation (Jacobson et al., 2016a; Krumrei-Mancuso, 2016), body alienation (Jacobson et al., 2013), and depersonalization (Jacobson et al., 2016b). Primarily small, positive links have been observed. Again, with some exceptions, such as body satisfaction not predicting decreased anxiety about one's aging appearance among older men and women (Homan & Boyatzis, 2009).

A potential explanation for the somewhat mixed results in this literature is that the implications of sanctification may differ based on how religious participants are or the particular religious beliefs they hold. For example, there is some suggestion that sanctification only promotes health behaviors among individuals who have a strong religious-orientated sense of control, meaning that the belief that people can work collaboratively with God to achieve their goals (Krause et al., 2017). For this reason, it is important to continue to examine the implications of sanctification appraisals across a variety of samples, including the general population, and to take into account other religious and spiritual factors.

Consistent with existing literature, we hypothesized that sanctifying one's body would be associated with greater body appreciation (H1). In addition, given evidence that better body image and body esteem are associated with better self-esteem (e.g., Morin et al., 2011; Tylka & Wood-Barcalow, 2015), we also hypothesized that sanctifying one's body would be associated with better self-esteem (H2). Further, we anticipated sanctification would predict variance in body appreciation and self-esteem beyond that accounted for by general religiousness and spirituality. Given gender differences in body appreciation (He et al., 2020) and nuanced gender differences in self-esteem (Gentile et al., 2009), we also conducted exploratory analyses to examine whether links between body sanctification and these outcomes would be moderated by gender.

### Sanctification of physical activity

Sport and religion share important cultural themes, such as beliefs, ritual, doctrine, sacrifice, relics, pilgrimage, and redemption (Fernández & Cachán-Cruz, 2014; Scholes & Sassower, 2014). Sport has even been classified as a civil religion (Forney, 2010). However, not much is known about the extent to which individuals sanctify, in a spiritual sense, their personal engagement in sport or physical activity. In an experimental study of sanctification of physical movement, participants were randomly assigned to ascribe sacred or nonsacred meaning to arm movements they were making (Freeburg et al., 2023). Those assigned to attribute sacred meaning to arm movements experienced more positive emotions and less negative emotions than those assigned to ascribe nonsacred meaning. This opens questions about the potential downstream effects of sanctification of physical activity for mental health. In addition, qualitative research suggests women can experience sacredness within and outside of themselves through dance and that dance can facilitate sanctification of the body (Leseho & Maxwell, 2010). Qualitative work has suggested belly dancing is associated with meditative states of connection with higher powers, universal energy, or deeper parts of the self (Krause, 2009). For some individuals, belly dancing exemplifies their relationship with a higher power or is experienced as a gift from God (Krause, 2009).

Our goal was to expand the limited knowledge of sanctification of physical activity by examining how commonly people sanctify physical activity such as exercise and sport. We hypothesized that sanctifying physical activity would be associated with engaging in more physical activity (H3) and receiving more enjoyment from physical activity (H4). We anticipated sanctification would predict variance in both engagement and enjoyment above that accounted for by general religiousness and spirituality. Further, we anticipated that if the sample included participants at extremely high levels of physical activity, we might observe curvilinear links such that sanctification of physical activity would promote a moderate approach to physical activity (exploratory).

### Sanctification of nature

Those adhering to nature religions often consider nature sacred (Taylor & Witt, 2006). People both in and outside of such religions can experience encounters with sacredness in nature (Naor & Mayseless, 2020), which can occur through mysticism or sanctification appraisals. Sanctification appraisals include interpreting beauty, the sublime, or the enduring cycles of nature as representing spiritual realities (Deal & Magyar-Russell, 2022; Johnson, 2002). Yet, little work has examined this process empirically. A rare empirical study on the outcomes of sanctifying nature found in three independent samples that sanctifying nature was associated with a greater willingness to sacrifice personal funds to protect the environment (Tarakeshwar et al., 2001). However, sanctifying nature was not associated with pro-environmental behaviors—such as recycling, carpooling, and volunteering.

To expand this work, we hypothesized that sanctifying nature would predict experiencing higher connectedness to nature (H5) and engaging in more intentional nature exposure (H6). We anticipated sanctification would predict variance in connectedness and exposure above that accounted for by general religiousness and spirituality. Further, given that many individuals face barriers to nature access, including a lack of natural environments in the areas where they live, minimal transportation options, and financial obstacles to spending time in nature, we sought to understand whether links between sanctifying nature and connectedness to nature/intentional nature exposure would be moderated by people's ability to access nature.

### The current study

Guided by cognitive appraisal theory, we replicated previous research on sanctification appraisals in the area of the body as a benchmark for extending this work to the underexamined domains of physical activity and nature. Our goal was to examine among a U.S. community sample the overall prevalence, demographic differences in prevalence, and key associations of sanctifying three aspects of life: one's body, physical activity, and nature. Demographic differences in general religiousness and spirituality are known. Greater religiousness/spirituality has been observed among older compared to younger individuals (Major-Smith et al., 2023; Zimmer et al., 2016); among other-than-White races and Hispanic ethnicities compared to non-Hispanic Whites (Major-Smith et al., 2023; Marquine et al., 2014; Taylor et al., 2009), and among those with higher educational attainment and income (Major-Smith et al., 2023). In the U.S., Republicans are more likely to identify as religious than Democrats (Newport, 2023). We wanted to explore if differences in sanctification would mirror these differences in general religiousness and spirituality. Knowledge about the prevalence of sanctification among particular demographic groups can be helpful background information for mental health professionals.

In addition, our primary aim was to examine how appraisals of sanctification in the domains of body, physical activity, and nature relate to indicators of mental and physical health. In doing so, we employed the two most commonly used measures of sanctification, which focus on (1) the extent to which participants view something as having sacred qualities and (2) the extent to which participants interpret something as a manifestation of a God or a Higher Power.

The hypotheses, procedures, and analyses were preregistered together with a qualitative companion study at https://osf.io/8bsxe. All procedures were performed in compliance with relevant laws and institutional guidelines, participants provided informed consent prior to participating, and their privacy rights were observed. This study was approved by Pepperdine University's Institutional Review Board (Protocol #: 21–06–1606; July 6, 2021).

## Method

### Materials

Items were averaged for each scale to create a scale score reflecting a greater amount of the construct. Internal consistency is reported in each case for the current sample.

#### Sacred qualities in the body, physical activity, and nature (nontheistic sanctification)

The Sacred Adjectives Scale (Mahoney et al., 2003) assessed the extent to which participants endorsed a list of sacred qualities about their bodies, physical activity (defined for participants as “intentional physical activity that noticeably increases your heart rate such as exercise or sport”), and nature. For each construct, 11 adjectives were rated on a 7-point scale tailored to the measure, for example, ranging from (1) “Does not at all describe my body” to (7) “Very closely describes my body”. The items can be seen in full in Table 1. Internal consistency was α = 0.93 for sacred adjectives of the body, α = 0.95 for sacred adjectives of physical activity, and α = 0.89 for sacred adjectives of nature.

**Table 1:** Items of the sacred qualities scales and percentage of participants who agreed, disagreed, or were neutral about each sacred quality representing body, physical activity, and nature (*N* = 342).

**How much does each adjective below describe how you think about your body?** **My body is…**				
	% Does not describe my body (anchors 1 – 3)	% Neutral (anchor 4)	% Describes my body (anchors 5 – 7)	*M* (*SD*)
Holy	39.8	27.5	32.7	3.65 (1.93)
Inspiring	46.8	26.0	27.2	3.38 (1.72)
Blessed	29.2	24.3	46.5	4.19 (1.97)
Sacred	33.6	23.7	42.7	4.03 (1.97)
Awe provoking	68.1	18.1	13.7	2.56 (1.68)
Heavenly	58.5	23.4	18.1	2.93 (1.89)
Spiritual	39.8	21.3	38.9	3.79 (2.07)
Religious	55.8	17.0	27.2	3.11 (2.06)
Mysterious	51.5	20.2	28.4	3.22 (1.83)
Miraculous	40.9	19.3	39.8	3.77 (2.08)
Divine	53.2	21.9	24.9	3.12 (2.00)

**How much does each adjective below describe how you think about physical activity that noticeably increases your heart rate (such as exercise or sport)?** **Physical activity is…**				
	% Does not describe physical activity (anchors 1 – 3)	% Neutral (anchor 4)	% Describes physical activity (anchors 5 – 7)	*M* (*SD*)
Holy	62.6	20.4	17.0	2.73 (1.87)
Inspiring	14.6	11.7	73.7	5.05 (1.77)
Blessed	50.0	20.5	29.5	3.56 (1.99)
Sacred	55.8	24.0	20.2	2.98 (1.87)
Awe provoking	38.0	19.0	43.0	3.83 (1.93)
Heavenly	60.5	20.8	18.7	2.85 (1.88)
Spiritual	47.7	15.5	36.8	3.47 (2.02)
Religious	68.7	15.8	15.5	2.49 (1.77)
Mysterious	62.3	18.7	19.0	2.80 (1.80)
Miraculous	45.3	18.7	36.0	3.49 (2.07)
Divine	57.6	21.3	21.1	2.92 (1.95)

**How much does each adjective below describe how you think about nature?** **Nature is…**				
	% Does not describe nature (anchors 1 – 3)	% Neutral (anchor 4)	% Describes nature (anchors 5 – 7)	*M* (*SD*)
Holy	24.6	20.5	55.0	4.63 (2.06)
Inspiring	2.6	3.2	94.2	6.28 (1.05)
Blessed	14.3	12.6	73.1	5.25 (1.87)
Sacred	10.8	12.9	76.3	5.58 (1.69)
Awe provoking	1.5	2.6	95.9	6.40 (0.98)
Heavenly	18.4	15.5	66.1	5.05 (1.98)
Spiritual	10.5	15.2	74.3	5.40 (1.68)
Religious	43.6	23.7	32.7	3.63 (2.08)
Mysterious	7.6	7.0	85.4	5.73 (1.51)
Miraculous	5.6	4.6	89.8	6.08 (1.34)
Divine	18.1	14.9	67.0	5.08 (1.98)

#### Manifestation of God in the body, physical activity, and nature (theistic sanctification)

The Manifestation of God Scale (Mahoney et al., 1999) assessed the degree to which participants perceived their bodies, physical activity (defined for participants as “intentional physical activity that noticeably increases your heart rate such as exercise or sport”), and nature as a manifestation of their beliefs and experiences of God. Items describe theocentric (God-centered) views, such as, “My body is a temple of God,” “I experience God through physical activity,” and “God is present in nature.” Items were rated on a 5-point scale ranging from (1) “Strongly disagree” to (5) “Strongly agree” (α = 0.99 for manifestation of God in the body, 12 items; α = 0.98 for manifestation of God in physical activity, 7 items; α = 0.98 for manifestation of God in nature, 9 items).

#### Body appreciation

The Body Appreciation Scale-2 (Tylka & Wood-Barcalow, 2015) assessed participants’ acceptance of, favorable opinions toward, and respect for their bodies. A sample item is, “I feel love for my body.” Items were rated on a 5-point scale ranging from (1) “Never” to (5) “Always” (α = 0.95, 10 items).

#### Self-esteem

The Rosenberg Self-esteem Scale (Rosenberg, 1965) assessed global self-worth. A sample item is, “I feel that I have a number of good qualities”. Items were rated on a 4-point scale ranging from (1) “Strongly disagree” to (4) “Strongly agree” (α = 0.93, 10 items).

#### Amount of physical activity

The Godin Leisure-Time Exercise Questionnaire assessed the amount of physical activity during participants’ leisure time (Godin & Shephard, 1997). The scale was used to create a physical activity score on the basis of the number of times a person engages in mild, moderate, and strenuous physical activity in a week (α = 0.73, 3 items).

#### Enjoyment from physical activity

The Physical Activity Enjoyment Scale assessed participants’ level of enjoyment derived from leisure-time physical activity (Mullen et al., 2011). Items were rated on a 7-point bipolar rating scale in which the first anchor reflected a lack of pleasure and the second anchor reflected greater pleasure, for example: (1) “It's very unpleasant” to (7) “It's very pleasant” (α = 0.96, 8 items).

#### Connectedness to nature

The Connectedness to Nature Scale assessed participants’ trait levels of feeling emotionally connected to the natural world (Mayer & Frantz, 2004). A sample item is, “I often feel a sense of oneness with the natural world around me.” Items were rated on a 5-point scale ranging from (1) “Strongly disagree” to (5) “Strongly agree” (α = 0.87, 14 items).

#### Intentional nature exposure

The Nature Exposure Scale II assessed participants’ intentional direct physical and/or sensory contact with the natural environment (Wood et al., 2019). Consistent with the authors’ recommendations, we administered items 2–6 of the original scale. A sample item is, “How much do you notice the natural environments in your everyday life?” Items were rated on a 5-point scale ranging from (1) “Low” to (5) “High” or from (1) “Not much” to (5) “A great deal”, depending on the item (α = 0.75, 5 items).

#### Access to nature

We created the 3-item access to natural environments index. The first item asked, “In your everyday life, if you wanted to walk in a natural environment (e.g., a park, forest, grass field, or beach), approximately how many miles would you have to travel from where you live to do this?” Participants enter a number representing miles. Responses were coded to represent 1 = 10 or more miles, 2 = 4 up to 10 miles, 3 = 2 up to 4 miles, 4 = 1 up to 2 miles, and 5 = <1 mile. The second item asked, “In your everyday life, how hard is it for you to physically access a natural environment in which you could walk (e.g., a park, forest, grass field, or beach)? Participants responded on a Likert scale consisting of (1) “Extremely difficult”, (2) “Somewhat difficult”, (3) “Neither easy nor difficult", (4) “Somewhat easy”, and (5) “Extremely easy”. The third item asked, “How many natural environments (e.g., a park, forest, grass field, or beach) do you have easy access to from where you live?” with response options including (1) “None”, (2) “One”, (3) “A few” and (4) “Many”. Responses were converted to z-scores before averaging into an index of access to nature (α = 0.69, 3 items).

#### Demographic and religious characteristics

Participants self-reported gender, race, ethnicity, education, and political affiliation. They rated their level of spirituality and religiousness with a single item each. We used the organizational religious activity and non-organizational religious activity items of the Duke University Religion Index (Koenig & Büssing, 2010) to assess religious attendance and private religious activity, respectively.

### Participants

English-speaking adults from the U.S. (*N* = 342) were recruited and paid through the research platform Prolific. In order to be included in the study, participants had to indicate that they were at least slightly spiritual or slightly religious. Participants who indicated they were neither religious nor spiritual were excluded. A majority of the sample identified as women (57.0 %), White or European American (77.8 %), and as having at least a bachelor's degree (59.6 %). Median household income was $50,000 USD. A majority of participants (63.7 %) indicated they were moderately or very spiritual. Fewer (36.3 %) were moderately or very religious. The religions represented included 45.3 % Protestant Christian, 12.3 % Roman Catholic, 0.6 % Orthodox Christian, 0.6 % Mormon, 1.5 % Jewish, 0.9 % Muslim, 0.9 % Hindu, 0.3 % Buddhist, and 5 % another religion (such as Paganism) or a combination of religions. Additionally, 14.9 % identified as having no religion, 13.7 % identified as agnostic, and 4.1 % identified as atheist. Table 2 reports the details of all demographics assessed.

**Table 2:** Descriptive information and demographic differences for sanctification of body, physical activity, and nature (*N* = 342).

	Total Sample	Sacred qualities body	Sacred qualities physical activity	Sacred qualities nature
	*n* ( %)	*M (SD)*	*M(SD)*	*M(SD)*
Overall sample (Possible range = 1–7)	342	3.43 (1.48)^b^	3.26 (1.56)^b^	5.37 (1.18)^a^
**Gender**				
Cisgender man	140 (40.9)	3.44 (1.45)	3.38 (1.49)	5.30 (1.16)
Cisgender woman	195 (57.0)	3.43 (1.50)	3.17 (1.60)	5.42 (1.20)
Transgender, nonbinary, genderfluid, nonconforming, or other	6 (1.7)	3.15 (1.80)	2.92 (1.37)	5.36 (0.95)

**Race and Ethnicity**				
White or European American	266 (77.8)	3.30 (1.41)	3.12 (1.49)	5.30 (1.21)
Black or African American	47 (13.7)	4.24 (1.52)	3.90 (1.71)	5.76 (1.06)
Asian or Asian American	25 (7.3)	3.42 (1.35)	3.61 (1.23)	5.23 (0.93)
Latinx or Hispanic American	13 (3.8)	2.51 (1.69)	2.66 (1.68)	5.62 (0.84)
American Indian or Alaskan Native	12 (3.5)	3.23 (1.70)	4.21 (1.53)	5.46 (1.14)
Native Hawaiian or Pacific Islander	1 (0.3)	3.09 (-)	2.91 (-)	5.73 (-)

**Education**				
Some high school	4 (1.2)	2.86 (1.49)	1.95 (0.69)	6.11 (0.63)
High school	134 (39.2)	3.48 (1.45)	3.36 (1.54)	5.47 (1.19)
Bachelor's degree	142 (41.5)	3.37 (1.52)	3.23 (1.59)	5.33 (1.13)
Graduate or professional degree	62 (18.1)	3.52 (1.48)	3.21 (1.55)	5.20 (1.26)

**Political affiliation**				
Democrat	146 (42.7)	3.18 (1.45)	3.32 (1.56)	5.31 (1.20)
Independent	107 (31.3)	3.65 (1.55)	3.34 (1.52)	5.50 (1.12)
Republican	62 (18.1)	3.58 (1.34)	3.02 (1.46)	5.35 (1.18)
Other/No preference	27 (7.9)	3.60 (1.54)	3.16 (1.68)	5.26 (1.30)

**Religiosity**				
Not religious at all ^a^	130 (38.00)	2.75 (1.31) ^b,c,d^	2.83 (1.43) ^b,d^	5.06 (1.21) ^c,d^
Slightly religious ^b^	88 (25.70)	3.43 (1.41) ^a,d^	3.47 (1.33) ^a^	5.42 (1.12)
Moderately religious ^c^	80 (23.40)	3.81 (1.30) ^a,d^	3.33 (1.54)	5.63 (1.05) ^a^
Very religious ^d^	44 (12.90)	4.78 (1.25) ^a,b,c^	3.99 (1.99) ^a^	5.75 (1.20) ^a^

**Spirituality**				
Not spiritual at all ^a^	6 (1.8)	2.24 (1.30) ^d^	1.89 (0.71) ^d^	4.42 (1.16) ^d^
Slightly spiritual ^b^	118 (34.5)	2.64 (1.19) ^c,d^	2.82 (1.30) ^d^	4.91 (1.22) ^c,d^
Moderately spiritual ^c^	119 (34.8)	3.44 (1.33) ^b,d^	3.19 (1.44) ^d^	5.46 (0.98) ^b,d^
Very spiritual ^d^	99 (28.9)	4.44 (1.37) ^a b,c^	3.96 (1.75) ^a,b,c^	5.87 (1.11) ^a,b,c^

**Organizational religious activity**				
Never^a^	151 (44.2)	2.94 (1.38) ^d,e,f^	2.94 (1.47) ^d,f^	5.19 (1.22)
Once a year or less^b^	67 (19.6)	3.43 (1.51) ^e,f^	3.40 (1.52)	5.40 (1.20)
A few times a year^c^	49 (14.3)	3.40 (1.29) ^e,f^	3.32 (1.35)	5.58 (1.05)
A few times a month^d^	19 (5.6)	4.33 (1.64) ^a^	4.07 (1.64) ^a^	5.62 (0.90)
Once a week^e^	39 (11.4)	4.37 (1.09) ^a, b,c^	3.40 (1.73)	5.46 (1.20)
More than once/week^f^	17 (5.0)	4.77 (1.18) ^a,b,c^	4.14 (1.91) ^a^	5.84 (1.11)

**Nonorganizational religious activity**				
Rarely/never ^a^	153 (44.7)	2.79 (1.34) ^b,d,e,f^	2.85 (1.42) ^e,f^	5.04 (1.24) ^d,e,f^
A few times a month ^b^	35 (10.2)	3.56 (1.46) ^a,f^	3.46 (1.45)	5.41 (0.98)
Once a week ^c^	14 (4.1)	3.83 (1.21)	3.94 (1.25)	5.82 (0.77)
Two or more times/week ^d^	43 (12.6)	3.61 (1.28) ^a,f^	3.37 (1.59)	5.70 (0.86) ^a^
Daily ^e^	52 (15.2)	4.05 (1.42) ^a^	3.59 (1.51) ^a^	5.60 (1.11) ^a^
More than once/day ^f^	45 (13.2)	4.52 (1.28) ^a,b,e^	3.79 (1.87) ^a^	5.78 (1.25) ^a^

	*M (SD)*	Possible Range		

Age in years	46.53 (16.09)	18+		
Body appreciation	3.42 (0.87)	1 −5		
Self-esteem	2.93 (0.67)	1–4		
Connectedness to nature	3.59 (0.70)	1–5		
Nature exposure	4.09 (0.76)	1–5		
Enjoyment of physical activity	4.50 (1.67)	1–7		
Amount of physical activity	13.26 (9.11)	N/A		

*Notes.* A repeated-measures ANOVA was conducted to compare levels of sanctification for the body, physical activity, and nature. Forms of sanctification that do not share superscripts differed significantly from one another following Bonferroni-adjustment. For categorical demographic variables significantly correlated with sanctification variables (see Table 2), ANOVAs were conducted to examine group differences. For each form of sanctification, levels of a demographic that do not share superscripts with the category superscripts in the first column, were significantly different following Bonferroni-adjustments. Participants were able to select more than one race/ethnicity, therefore comparisons between racial and ethnic groups were not conducted. Table 2 displays which racial and ethnic identifications were associated with more or less sanctification of body, physical activity, and nature compared to all other racial and ethnic identifications.

*p < .05., **p < .01, *** *p* < .001.

### Procedure

Surveys were counterbalanced and administered via Qualtrics. To increase reliability, we deleted listwise participants who at the end of the survey indicated, when asked without the risk of negative consequences, that they had not been attentive or honest in their responses (*n* = 4) and participants who failed an attention check (*n* = 2). We excluded participants who were outliers on variables per analysis, using the outlier labeling rule (Hoaglin et al., 1986) with a conservative 2.2. multiplier (*n* = 8 for amount of physical activity and *n* = 1 for nature connectedness).

## Results

### Links between theistic and nontheistic sanctification of body, physical activity, and nature

Theistic and nontheistic forms of sanctification were highly correlated with one another: the correlation between sacred qualities of the body and manifestation of God in the body was *r* = 0.64 (*p* < .001), the correlation between sacred qualities of exercise and manifestation of God in exercise was *r* = 0.61 (*p* < .001), and the correlation between sacred qualities of nature and manifestation of God in nature was *r* = 0.50 (*p* < .001).

Our preregistered plan was to report findings for both theistic and nontehistic sanctification of the body, nature, and physical activity, as individual predictors of our outcomes of interest. Given the relatively high correlation between the two forms of sanctification and the largely parallel findings that emerged for the two forms of sanctification, we responded to reviewers’ requests to streamline the paper by deciding to present in full detail the analyses for nontheistic sanctification (i.e., sacred qualities) and reporting in the table notes any differences in significance in the findings involving the Manifestation of God scales compared the findings involving the Sacred Qualities scales. We opted to present in detail the results of the Sacred Qualities scales given that these scales are a bit more widely applicable to the general population than the Manifestation of God scales, given that sacred qualities can be endorsed by theists and nontheists alike, including spiritual‑but-not-religious individuals. In contrast, the manifestation of God scales are typically endorsed only by theists.

### Descriptive information about sanctification and demographic differences for sanctification of body, physical activity, and nature

Table 1 displays the percentage of participants who agreed, disagreed, or were neutral about whether each sacred quality represented their body, physical activity, and nature. Table 2 displays the overall means for sanctification of the body, physical activity, and nature. A repeated-measures ANOVA with Bonferroni-adjustment indicated that participants were more likely to sanctify nature than either their bodies or physical activity. There were no significant differences in the degree to which participants sanctified their bodies and physical activity (*p* = .07).

To explore demographic differences in sanctification, we conducted point-biserial and Pearson correlations between demographic variables and sanctification of the body, physical activity, and nature (see Table 3). As participants could identify with more than one race or ethnicity, we created binary categories based on those who did and did not identify with each racial and ethnic category. Identifying as White was associated with less sanctification of the body, exercise, and nature. Conversely, identifying as Black was associated with more sanctification of the body, exercise, and nature. Identifying as Latinx was associated with sanctifying one's body less. Identifying as American Indian or Alaskan Native was associated with sanctification nature more. However, note the small group sizes for Latinx (*n* = 13) and American Indian or Alaskan Native (*n* = 12). Age was associated with sanctifying one's body more. Gender, education, income, and political affiliation were not associated with sanctification. Religiousness, spirituality, and nonorganizational religious activity predicted greater sanctification in all domains and organizational religious activity predicted greater sanctification of the body and exercise.

**Table 3:** Point-biserial and pearson correlations between demographic variables and sanctification variables (*N* = 342).

	Sacred qualities body	Sacred qualities physical activity	Sacred qualities nature
Cisgender man vs cisgender woman	.01	.07	−0.05
White vs other	−0.16**	−0.17**	−0.12*
Black vs other	.22***	.17**	.13*
Asian or Asian American vs other	−0.00	−0.04	.06
Latinx vs other	−0.12*	−0.08	.04
American Indian or Alaskan Native	−0.03	.01	.12*
Native Hawaiian or Pacific Islander	−0.01	.02	−0.01
Education	0.1	−0.01	−0.10
Republican vs democrat	.13	−0.09	.02
Age	.11*	.04	−0.03
Income	−0.04	.02	.07
Religiousness	.45***	.22***	.22***
Spirituality	.49***	.31***	.34***
Organizational religious activity	.38***	.19***	.14**
Nonorganizational religious activity	.42***	.22***	.25***

*Note.* Based on group sizes, only those who identified as a cisgender man or cisgender woman were included in gender comparisons. Parallel correlations were also run for manifestation of God in the body, physical activity, and nature. The pattern of significance and directionality of the correlations was the same for the manifestation of God scales as for the sacred qualities scales with the following exceptions: (a) the correlation between Latinx and sanctification of the body was not significant when using the manifestation of God scale, (b) significant correlations emerged for Republicans versus Democrats and manifestation of God in the body (*r* = 0.32***), physical activity (*r* = 0.17*), and nature (*r* = 0.25***), suggesting that Republicans are more likely than Democrats to view God as manifested in these aspects of life, and (c) whereas age was only correlated with sacred qualities of the body, age was correlated with manifestation of God in the body (*r* = 0.23***), physical activity (*r* = 0.17**), and nature (*r* = 0.20***).

*p < .05., **p < .01, *** *p* < .001.

### Sanctification as a predictor of indicators of mental and physical health

To test our hypotheses, we conducted six hierarchical regressions. All continuous predictors were grand-mean centered. For all regressions, we had made the a priori decision based on previous research to enter as controls gender, race, ethnicity, age, and income in Step 1. In Step 2, we entered religiousness, spirituality, organizational religious activity, and nonorganizational religious activity, in order to examine whether sanctification would predict outcomes above and beyond being religious or spiritual in general. In Steps 3, we entered the relevant sanctification variable (body, physical activity, or nature). Finally, we included a fourth step when assessing a moderating effect of gender or nature access. The results are reported in Table 4.

**Table 4:** Sanctification predicting outcome variables.

**Regression 1: Body Appreciation (*n* = 335)**				
	**β**	***R^2^_adj_***	ΔR^2^	ΔF
**Step 1**		.11	.12	7.67**
Man vs woman	.10			
White vs other	−0.11			
Black vs other	.19*			
Latinx vs other	−0.02			
Age	.20**			
Income	.11*			
**Step 2**		.14	.04	4.05**
Religiosity	.04			
Spirituality	.16*			
Organizational Religious Activity	−0.02			
Nonorganizational Religious Activity	.06			
**Step 3**		.26	.12	51.52**
Sacred Qualities Body	.42**			
**Step 4**		.26	.003	1.43
Gender X Sacred Qualities Body	.08			

**Regression 2: Self-Esteem (*n* = 335)**				
	**β**	***R^2^_adj_***	**Δ*R^2^***	**ΔF**
**Step 1**		.19	.20	13.94**
Man vs woman	.11*			
White vs other	−0.04			
Black vs other	.09			
Latinx vs other	−0.01			
Age	.43**			
Income	.07			
**Step 2**		.20	.02	2.15
Religiosity	.10			
Spirituality	.04			
Organizational Religious Activity	.02			
Nonorganizational Religious Activity	.01			
**Step 3**		.24	.04	18.48**
Sacred Qualities Body	.25*			
**Step 4**		.24	.00	0.16
Gender X Sacred Qualities Body	−0.03			

**Regression 3: Amount of Physical Activity (*n* = 334)**				
	**β**	***R^2^_adj_***	**Δ*R^2^***	**ΔF**
**Step 1**		0.03	0.05	2.65*
Man vs woman	.11*			
White vs other	−0.04			
Black vs other	−0.08			
Latinx vs other	−0.11			
Age	−0.14*			
Income	.03			
**Step 2**		0.02	0.00	0.36
Religiosity	−0.01			
Spirituality	.03			
Organizational Religious Activity	.03			
Nonorganizational Religious Activity	.04			
**Step 3**		0.04	0.02	5.91*
Sacred Qualities Physical Activity	.14*			

**Regression 4: Enjoyment of Physical Activity (*n* = 342)**				
	**β**	***R^2^_adj_***	**Δ*R^2^***	**ΔF**
**Step 1**		0.04	0.05	3.06**
Man vs woman	.12*			
White vs other	.04			
Black vs other	.10			
Latinx vs other	−0.13*			
Age	.04			
Income	.08			
**Step 2**		0.3	0.01	0.68
Religiosity	−0.09			
Spirituality	.03			
Organizational Religious Activity	.07			
Nonorganizational Religious Activity	.06			
**Step 3**		0.13	0.09	36.05**
Sacred Qualities Physical Activity	.33**			

**Regression 5: Connectedness to Nature (*n* = 341)**				
	**β**	***R^2^_adj_***	**Δ*R^2^***	**ΔF**
**Step 1**		0.02	0.04	2.17*
Man vs woman	−0.09			
White vs other	.08			
Black vs other	.07			
Latinx vs other	−0.04			
Age	.10			
Income	.11*			
**Step 2**		0.16	0.15	14.30**
Religiosity	−0.34**			
Spirituality	.35**			
Organizational Religious Activity	−0.08			
Nonorganizational Religious Activity	.02			
**Step 3**		0.29	0.13	30.88**
Sacred Qualities Nature	.37*			
**Step 4**		0.29	0.00	0.02
Nature Access X Sacred Qualities Nature	.03			

**Regression 6: Intentional Nature Exposure (*n* = 342)**				
	**β**	***R^2^_adj_***	**Δ*R^2^***	**ΔF**
**Step 1**		0.02	0.04	2.00
Man vs woman	.02			
White vs other	.13			
Black vs other	−0.01			
Latinx vs other	−0.04			
Age	.08			
Income	.06			
**Step 2**		0.05	0.04	3.66**
Religiosity	−0.20**			
Spirituality	.09			
Organizational Religious Activity	.03			
Nonorganizational Religious Activity	.19*			
**Step 3**		0.20	0.16	32.63**
Sacred Qualities Nature	.17**			
**Step 4**		0.21	0.01	4.53*
Nature Access X Sacred Qualities Nature	−0.51*			

*Notes.* Due to small subgroups for other gender identities, only self-reported cisgender men and women were included for analyses involving gender. Parallel regressions were run for manifestation of God in the body, physical activity, and nature. The pattern of significance and directionality were the same for the Manifestation of God scales as for the Sacred Qualities scales with the following three exceptions: (a) gender weakly moderated the relationship between manifestation of God in the body and body appreciation; for both women and men, experiencing the body as a manifestation of God predicted greater body appreciation, but this relationship was slightly stronger in men than in women, (b) manifestation of God in the body did not predict greater self-esteem, and (c) manifestation of God in nature did not predict greater intentional nature exposure.

* *p* < .05, ** *p* < .01.

#### The relationship between sanctification of the body and body appreciation and self-esteem

H1 was that sanctifying the body would be associated with greater body appreciation, beyond the effects of general religiousness or spirituality. We also explored whether there would be an interaction between sanctification of the body and gender. As seen in Table 4 (Regression 1), the variables accounted for variation in participants’ body appreciation. Identifying as Black, age, income, and spirituality were small predictors of greater body appreciation. Even beyond the variance attributed to demographic factors and general religiousness and spirituality, viewing the body as having sacred qualities moderately predicted greater body appreciation. Gender did not moderate the relationship between viewing the body as having sacred qualities and body appreciation.

We conducted a regression to examine H2 that sanctifying one's body would be associated with greater self-esteem, beyond the effects of general religiousness or spirituality. As seen in Table 4 (Regression 2), the variables accounted for variation in participants’ self-esteem. Being male was a weak and age a moderate predictor of greater self-esteem. Even beyond the variance attributed to demographic factors and general religiousness and spirituality, viewing the body as having sacred qualities weakly predicted greater self-esteem. This relationship was not moderated by gender.

#### The relationship between sanctification of physical activity and engagement in/enjoyment of physical activity

We conducted a regression to examine H3 that sanctifying physical activity would be associated with engaging in more physical activity, beyond the effects of general religiousness or spirituality. As seen in Table 4 (Regression 3), the variables accounted for variation in participants’ physical activity. Identifying as male and age were weak predictors of engaging in more physical activity. Even beyond the variance attributed to demographic factors and general religiousness and spirituality, viewing physical activity as having sacred qualities weakly predicted engaging in more physical activity.

We conducted exploratory analyses to determine whether sanctifying physical activity would result in taking a moderate approach to physical activity: that is, not engaging in too little *or too much* sport or exercise. For the analyses, we repeated Regression 3 with an additional step including quadratic sanctification of physical activity. Even when including outliers for physical activity, the parabolic effects of viewing physical activity as having sacred qualities (Δ *R^2^* < 0.001, *p* = .94) did not account for additional variability in physical activity.

We conducted a regression to examine H4 that sanctifying physical activity would be associated with greater enjoyment of physical activity, beyond the effects of general religiousness or spirituality. As seen in Table 4 (Regression 4), the variables accounted for variation in participants’ enjoyment of physical activity. Identifying as Latinx weakly predicted less and identifying as male weakly predicted more enjoyment of physical activity. Even beyond the variance attributed to demographic factors and general religiousness and spirituality, viewing physical activity as having sacred qualities moderately predicted enjoying physical activity more.

#### The relationship between sanctification of nature and connectedness to nature and intentional nature exposure

We conducted a regression to examine H5 that sanctifying nature would be associated with experiencing greater connectedness to nature, beyond the effects of general religiousness or spirituality. In this regression, we also examined the interaction between sanctification of nature and access to nature. As seen in Table 4 (Regression 5), the variables accounted for variation in participants’ levels of connectedness to nature*.* Income was a weak predictor of greater connectedness to nature. Religiousness was a moderate predictor of less connectedness to nature and spirituality was a moderate predictor of more connectedness to nature. Even beyond the variance attributed to demographic factors and general religiousness and spirituality, viewing nature as having sacred qualities moderately predicted greater connectedness to nature. Nature access did not moderate this relationship.

We conducted a regression to examine H6 that sanctifying nature would be associated with greater intentional nature exposure, beyond the effects of general religiousness or spirituality. In this regression, we also examined the interaction between sanctification of nature and access to nature. As seen in Table 4 (Regression 6), the variables accounted for variation in participants’ levels of intentional nature exposure. Religiousness moderately predicted less intentional nature exposure and spirituality moderately predicted more intentional nature exposure. Even beyond the variance attributed to demographic factors and general religiousness and spirituality, viewing nature as having sacred qualities weakly predicted more intentional nature exposure, and nature access strongly moderated this relationship.

We post-hoc probed this moderation (Holmbeck, 2002) and found that for individuals with high access to nature, viewing nature as having sacred qualities was not associated with intentional nature exposure, *t*(338) = 1.16, *p* = .25. However, for those with low access to nature, viewing nature as having sacred qualities predicted engaging in more intentional nature exposure, *t*(338) = 3.93, *p* < .001.

## Discussion

The current study sought to examine the relationship between sanctification appraisals and indicators of mental and physical health. This work replicated past research examining sanctification appraisals of the body and extended this work to the lesser explored domains of nature and physical activity. Collectively, the results indicate sanctification is a noteworthy cognitive mechanism with weak-to-moderate associations with a range of beneficial attitudes and behaviors.

Although our write-up focused primarily on nontheistic sanctification as assessed by the Sacred Qualities scales, analyses were also conducted for theistic sanctification as assessed by the Manifestation of God scales (reported in the table notes). This multi-measure approach indicated a highly similar pattern of results for both theistic and nontheistic sanctification, offering replication of most of the observed relationships.

### Descriptive information

First, how common is it for individuals to sanctify their bodies, physical activity, and nature? Our sample of adults in the U.S. who were at least slightly spiritual or religious was most likely to sanctify nature, with mean scores for sacred qualities approaching “very closely describes” nature. They sanctified their bodies and physical activity to a lesser extent, where mean scores were closer to “neutral”. Given that over a dozen previous studies have been conducted on sanctification of the body, we now have an understanding that individuals may sanctify physical activity to similar levels and nature to much higher levels. The most common positively endorsed sacred qualities (percentage of the sample who endorsed the sacred quality above neutral) for the body were blessed, sacred and miraculous; for physical activity were inspiring, awe provoking, and spiritual; and for nature were awe provoking, inspiring, and miraculous. This provides a starting point for clinicians who desire to further understand whether and how individuals sanctify particular aspects of life.

As mental health professionals consider the potential relevance of sanctification to client's lives, it can also be helpful to be aware of demographic links. We observed, as might be expected, that being more religious or spiritual and engaging in more religious and spiritual activities were all associated with a greater likelihood of forming appraisals of sanctification. Further, consistent with demographic surveys indicating that Black Americans report higher religiousness and spirituality than White Americans (Pew Research Center, 2024), participants in our sample who identified as Black reported higher sanctification than other races and participants who identified as White reported lower sanctification than other races. In addition, consistent with previous work suggesting that people become more religious/spiritual as they age (Zimmer et al., 2016), older participants in our sample tended to endorse more sanctification appraisals than younger participants. Although we did not observe political differences based on our analyses using the Sacred Qualities measures, we observed that Republicans were more likely than Democrats to endorse that God was manifest in their bodies, physical activity, and nature. Overall, this demographic knowledge can be helpful for mental health professionals when considering the potential relevance of sanctification in clients’ lives.

### Sanctification of the body

Our hypothesis (H1) that body sanctification would be associated with greater body appreciation even after accounting for general religiousness/spirituality was supported. Viewing the body as having sacred qualities moderately predicted greater body appreciation. Our results support previous work showing links between sanctification and greater body connectedness, body satisfaction, body appreciation, and body esteem (Mahoney et al., 2022). Cross cultural qualitative research has demonstrated the role of sanctification for girls and women who report higher positive body image in interviews, and individuals who sanctify their bodies are more likely to care for and nourish their bodies (Wood-Barcalow et al., 2010).

Within the body image literature, stronger emphasis has historically been placed on the predictors of negative body image; however more recently there has been an increased focus on positive body image (Tylka & Huellemann, 2023; Tylka & Wood-Barcalow, 2015). Sanctification is a useful construct for broadening our understanding of the mechanisms that promote positive body image and body appreciation and could advance current prevention and intervention efforts to enhance body image for both men and women. For example, clinicians can consider interventions that embrace and support clients’ cognitive appraisals of sanctification when clients possess worldviews consistent with this approach. In one experiment, college women who read Christian-based affirmations that emphasized God's love and acceptance of their bodies increased in positive feelings about their appearance when subsequently viewing photos of ‘thin ideal’ fashion models compared to women in a control group who read statements about campus life (Boyatzis et al., 2007). This suggests women's beliefs and feelings about their physical appearance can be improved by being exposed to a vision of their bodies as divinely loved and accepted. Given the current emphasis on sacred qualities, it would be useful to think about interventions that reinforce sacred adjectives of the body, to extend previous work to individuals who are religious but not theists or individuals who are spiritual but not religious. More general approaches along these lines have been supported by meta-analyses (Bouwhuis-Van Keulen et al., 2024). Further, given that gender did not moderate the relationship between sanctifying one's body and body appreciation, it would be useful to tailor interventions not only to women, but also to men.

Our hypothesis (H2) that body sanctification would be associated with higher self-esteem even after accounting for general religiousness/spirituality was also supported. This builds on previous findings suggesting that positive body image and body esteem are associated with better self-esteem (Morin et al., 2011; Tylka & Wood-Barcalow, 2015). Previous research has suggested that sanctifying other aspects of life is associated with greater self-esteem (e.g., Doehring et al., 2009), however, this is the first study, to our knowledge, to examine the relationship between body sanctification and self-esteem. Although the observed effect size was small, the current findings encourage further exploration of this topic. If the link between sanctification of the body and self-esteem is replicated in additional work, this might encourage clinicians to consider how themes related to sanctification of the body, and perhaps by extension sanctification of self, may be useful for clients struggling with low self-esteem.

### Sanctification of physical activity

Previous links have been observed between sanctifying one's body and engaging in more exercise (Homan & Boyatzis, 2010; Mahoney et al., 2005), but almost no work has focused on understanding the outcomes of sanctifying physical activity itself. Our hypotheses that sanctification of physical activity would be associated with engaging in more physical activity (H3) and enjoying physical activity more (H4), even after accounting for general religiousness and spirituality, were supported. The link for engaging in more physical activity was weak and the link for enjoying physical activity was moderate. Future research may examine with longitudinal data whether it is the enjoyment of physical activity that functions as the reason that sanctification of physical activity is associated with engaging in more physical activity.

The current results enhance our understanding of the factors that may motivate participation in physical recreation, sports, and exercise. This can have meaningful implications for individuals in need of more movement and overall positive health and mental health effects. Interventions may be able to help people find meaning and enjoyment in moving their bodies by exploring the sacred aspects of physical activity or by finding unique ways to connect with something awe provoking, spiritual, or blessed through movement. Individuals who gravitate toward this way of thinking are more likely to engage in regular physical activity and find it enjoyable (Greenwood & Delgado, 2013).

### Sanctification of nature

Nature emerged as the most commonly sanctified domain in this study. Our hypothesis (H5) that nature sanctification would predict higher connectedness to nature even after accounting for general religiousness/spirituality was supported, with a moderate effect size. Previous work has demonstrated that people view nature as sacred (Deal & Magyar-Russell, 2022; Naor & Mayseless, 2020), and the current study builds on this work by indicating that individuals who do so feel more connected to nature. While this may not be surprising, this is the first study, to our knowledge, to empirically examine this.

Connectedness to nature is associated with a number of positive outcomes, including body appreciation (Swami, Barron et al., 2016), self-esteem (Swami, von Nordheim et al., 2016), and physical activity (Bacevičienė et al., 2021). Therefore, efforts to cultivate or capitalize on existing nature sanctification via prevention or intervention programs may benefit individuals in a wide variety of ways.

Our hypothesis (H6) that nature sanctification would predict higher intentional nature exposure even after accounting for general religiousness/spirituality was also supported. Despite the small effect size, this information can still serve useful given the link between time spent in nature and well-being (Bowler et al., 2010; McMahan & Estes, 2015; Norwood et al., 2019; Trammell et al., 2024). Additionally, it has been documented that nature exposure is linked to higher levels of body appreciation (Swami, 2024) and the physical benefits of exercise are heightened when one exercises outdoors (Boere et al., 2023). Therefore, similar to connectedness to nature, any entryway for increasing nature exposure may be beneficial to individuals in a broad range of ways.

However, consideration needs to be given to the fact that nature access is not equal for all individuals. This has implications for the outcomes we examined. Our exploratory analyses revealed that nature access strongly moderated the relationship between sanctification and intentional nature exposure. It was only individuals who reported low access to nature who were more likely to engage in intentional nature exposure when they viewed nature as having sacred qualities. This illustrates how sanctification can be an organizing force in people's lives, motivating them to overcome obstacles in order to engage with things they consider sacred. This provides valuable insight for clinicians who may want to encourage their clients to spend time in nature. Clients who have less access to nature, may be more likely to overcome these barriers if or when they are in tune with a sense of sacredness in nature.

### Limitations and future directions

The current work has resulted in an exploration of how individuals interpret the sacred qualities used to assess sanctification (Krumrei-Mancuso et al., 2025). Additional work is needed to replicate the current findings in the understudied areas of physical activity and nature, and to attend to the potential differences suggested by the current findings between theistic and nontheistic sanctification of nature in relation to intentional nature exposure. It would also be helpful for future work to examine more diverse samples, particularly with regard to religion and spirituality. Perhaps most importantly, longitudinal work is needed to better understand the temporal relationship between sanctification and outcomes of interest in the areas of health and wellbeing.

### Conclusion

Psychological research often defines religion or spirituality as self-identified categories that don't provide nuanced information about people's specific beliefs and behaviors. The study of sanctification and how it infuses people's lives provides a richer understanding of previously established links between religion/spirituality and mental/physical health (Abdel-Khalek et al., 2019; Garssen et al., 2021; Shattuck & Muehlenbein, 2020). The fact that sanctification predicted our outcome variables even after controlling general levels of religiousness/spirituality, suggests the importance for mental health professionals to not only be aware of general information such as the religious/spiritual affiliation of their clients, but to explore with clients for whom the sacred domain is salient the nuances of their religious/spiritual cognitions.

## Declaration of competing interest

The authors declare that they have no known competing financial interests or personal relationships that could have appeared to influence the work reported in this paper.
